# Lymphangiosarcoma of the arm presenting with lymphedema in a woman 16 years after mastectomy: a case report

**DOI:** 10.4076/1757-1626-2-6887

**Published:** 2009-09-01

**Authors:** Yasir J Sepah, Masood Umer, Asim Qureshi, Shaista Khan

**Affiliations:** Department of Surgery (Orthopedics), Aga Khan University Medical CollegeP.O. Box 3500, Karachi-74800Pakistan

## Abstract

Lymphangiosarcoma following breast cancer is a relatively rare entity, with around 300 cases so far reported worldwide. Affecting the long term survivors of breast cancer, lymphangiosarcoma (Stewart-Traves Syndrome) has a high mortality rate. Since lympedema following radical mastectomy or axillary clearance and radiotherapy seems to be the main predisposing factor, further research regarding modifications in the surgical technique of axillary nodes dissection as well as the development of new chemotherapeutic agents effective in lymphangiosarcoma are required.

## Introduction

Lymphangiosarcoma following breast cancer is a rare entity. Around 300 cases have been reported worldwide since Stewart-Traves syndrome characterized by angio sarcoma of skin and soft tissue of the area after Radical Mastectomy, Radiation and Lymphedema [1]. Its incidence in white American women is estimated to be 1.6 per 100,000 with only 25% of these sarcomas originating in the upper limb [2]. Lymphangiosarcoma is characterized by skin changes in the form of purple colored raised cutaneous lesions progressing to ulceration in a lymphedematous arm within a median of ten years following mastectomy [3]. Lymphangiosarcoma has a poor prognosis [4,5], with a 5 year survival of <5% with multimodality treatment. Wide surgical resection is the treatment of choice [4,6]. Several hypotheses have been postulated about its pathogenic mechanism. Lymphatic blockage, resulting in stimuli for growth factors and cytokines with a consequent proliferation of vessels and lymphatics is one hypothesis that explains the proliferation of lymphatic vessels in the affected edematous tissue [7]. The role of radiotherapy in the development of lymphangiosarcoma is questionable because most post radiation chest wall sarcomas are not lymphangiosarcomas [8]. Review of literature suggests that mastectomy resulting in Breast Cancer Related Lymphedema (BCRL) is the main predisposing factor [8]. Other factors such as cardiovascular diseases and hypertension have also been described in relation to lymphangiosarcoma.

## Case presentation

A 74-year-old lady with a history of hypertension and known case of breast cancer presented to our clinic with a swelling of the left proximal arm for the past three years. Patient was of Punjabi by ethnicity and was a Pakistani national.

**Figure 1.**
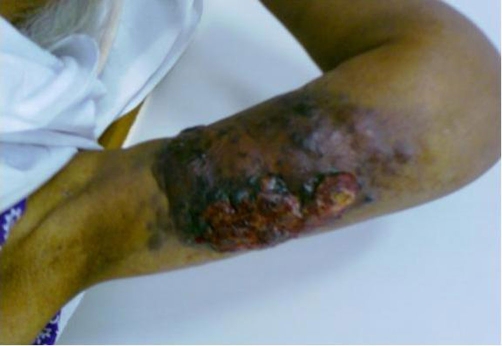
Image of the patient’s left arm showing a 10 × 12 cm lesion on the ulnar aspect of the arm.

**Figure 2.**
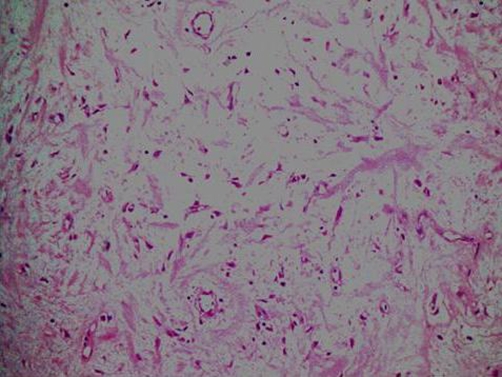
A low power view 20× magnification showing thin walled vascular channels lined by endothelial cells.

**Figure 3.**
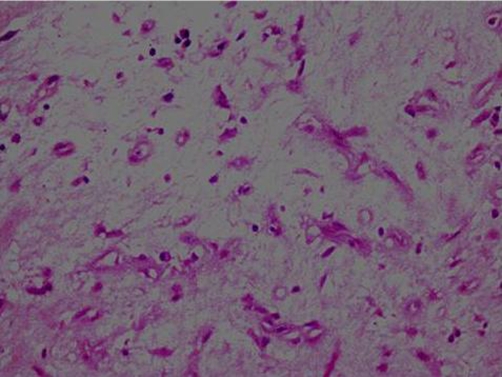
40× magnification showing vascular channels lined by endothelial calls showing nuclear atypia and pleomorphism. The surrounding stroma is loose and myxoid.

**Figure 4.**
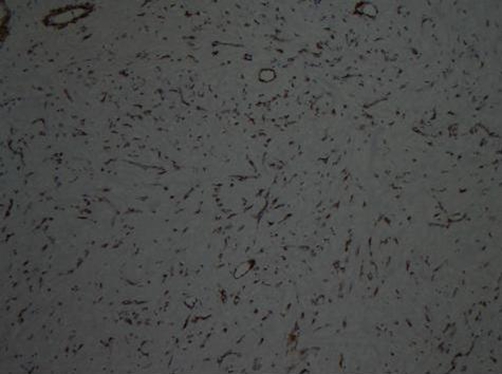
CD 31 immunohistochemical stain showing positivity in endothelial cells of blood vessels.

**Figure 5.**
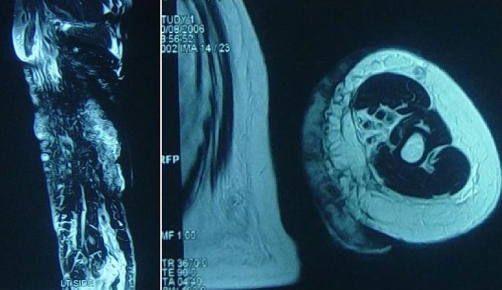
MRI of the left arm of the patient showing extension of the lesion marked with arrows.

She had under gone a modified radical mastectomy (MRM) 16 years ago in 1990 for infiltrating ductal carcinoma of the left breast, received 25 fractions of radiotherapy, followed by Tamoxifen for five years. The patient had a history of gradual increase in the size of the swelling upper arm and complained of non-radiating, mild to moderate pain with appearance of nodular lesions along antero medial aspect of upper arm (Figure 1); that had ulcerated in the proceeding 8 weeks. There was no history of fever, numbness and paresthesias in the left upper limb. On examination the patient was afebrile; with lymphedema of entire left arm. A 12 × 6 cm area of multiple ulcerated lesions with surrounding erythema and induration and absence of deep infiltration was noted. Motor power and range of motion was normal both at the elbow and shoulder joint. Biopsy from the mass confirmed the diagnosis of lymphangiosarcoma (Figures 2 and 3). A lymphovascular invasive pattern was seen and immunohistochemistry was positive for endothelial cell markers (Figure 4) (CD 31-CD36). There was no evidence of distant metastasis on metastatic workup. CXR showed bilateral pleural thickening more marked on the left side. CT scan of chest revealed thickening of the skin overlying the mass and non specific ground glass appearance in the upper lung fields bilaterally. No active pulmonary lesion was seen. Ultrasound liver and gallbladder was also normal. MRI showed a soft tissue mass occupying the medial side of the left upper arm with evidence of extension through the sub-coetaneous tissue and up to the muscle layer with enhancement (Figure 5), with absence of osseous disease on bone scan. Disarticulation of the left shoulder joint was performed successfully. The patient developed metastases in the brain and lungs 5 months after the procedure and expired a month later.

## Discussion

Currently there are 2 million breast cancer survivors in the United States alone and 20% of them suffer from Breast Cancer Related Lymphedema [9]. Advances in diagnostic aids, discoveries of newer chemotherapeutic agents and achievements of excellence in the field of surgery has offered substantial improvement in long term survival. This, however over the last 7 decades has been coupled with increasing number of complications as reported in literature [10]. BCRL leading to lymphangiosarcoma is one such rare complication. With an incidence of 10% it is estimated that United States alone will have 200,000 new cases of BCRL diagnosed every year [11,12]. Numerous studies have implicated lack of awareness (both on the part of the surgeon/physician and patient) in the ever increasing numbers of breast cancer survivors being diagnosed with BCRL [10,11,13]. Lack of consensus on the diagnostic criteria also seems to be one factor affecting an early diagnosis of this entity [11]. Although in majority of the cases reported so far postmastectomy, complete axillary node dissection and radiotherapy seem to be the risk factors for developing BCRL progressing to lymphangiosarcoma, there have also been reports of idiopathic lymphedema related to lymphangiosarcoma as well [4,14].

Strategies to decrease chronic lymphedema include manual and sequential pneumatic lymphatic compression [15,16], periodic examination [17], modification of the surgical technique [12], manual lymph drainage, compression garments, prescribed exercises and ultrasound therapy. More recently, use of Coumarin, intra-arterial injections of autologous lymphocytes, liposuction, Selenium, and laser treatment are also reported [16]. Advocated management of lymphangiosarcoma includes early diagnosis and radical surgical resection [6,8]. However, other promising options that need to be explored further, include radio-immunotherapy (54 Gy/Interferon beta) [18], doxorubicin which has shown promising results both in animal models and humans [19], isolated limb perfusion (ILP) tumor necrosis factor (TNF) and melphalan. Early diagnosis and conservative surgery of breast and axilla, has offered remarkable reduction incidence of lymphedema. Total preservation of axillary lymphatic with advent and extension of sentinel node biopsy, would further reduce this complication.

## Conclusion

Stewart-Traves syndrome is a rare entity with poor response to chemo therapy. Awareness about the pathology and its preceding sign & symptoms among both physicians and breast cancer survivors will ensure regular long term follow up resulting in an early diagnosis. Since lympedema following radical mastectomy or axillary clearance and radiotherapy seems to be the main predisposing factor, further research regarding modifications in the surgical technique of axillary nodes dissection as well as the development of new chemotherapeutic agents effective in Lymphangiosarcoma are required.

## Abbreviations

BCRL: breast cancer related lymphedema
ILP: isolated limb perfusion
MRM: modified radical mastectomy
